# Bone Marrow-Derived Multipotent Stromal Cells Promote Myocardial Fibrosis and Reverse Remodeling of the Left Ventricle

**DOI:** 10.1155/2015/746873

**Published:** 2015-01-21

**Authors:** Timur Fatkhudinov, Galina Bolshakova, Irina Arutyunyan, Andrey Elchaninov, Andrey Makarov, Evgeniya Kananykhina, Oksana Khokhlova, Arkady Murashev, Valeria Glinkina, Dmitry Goldshtein, Gennady Sukhikh

**Affiliations:** ^1^Research Center for Obstetrics, Gynecology and Perinatology of Ministry of Healthcare of the Russian Federation, 4 Oparina Street, Moscow 117997, Russia; ^2^Scientific Research Institute of Human Morphology, Russian Academy of Medical Sciences, 3 Tsurupa Street, Moscow 117418, Russia; ^3^Pirogov Russian National Research Medical University, Ministry of Healthcare of the Russian Federation, 1 Ostrovitianov Street, Moscow 117997, Russia; ^4^Biological Testing Laboratory, Branch of Shemyakin-Ovchinnikov Institute of Bioorganic Chemistry, Russian Academy of Sciences, 6 Nauki Avenue, Pushchino 142290, Russia; ^5^Research Centre of Medical Genetics of the Russian Academy of Medical Sciences, 1 Moskvorechie Street, Moscow 115478, Russia

## Abstract

Cell therapy is increasingly recognized as a beneficial practice in various cardiac conditions, but its fundamentals remain largely unclear. The fates of transplanted multipotent stromal cells in postinfarction cardiac microenvironments are particularly understudied. To address this issue, labeled multipotent stromal cells were infused into rat myocardium at day 30 after myocardial infarction, against the background of postinfarction cardiosclerosis. Therapeutic effects of the transplantation were assessed by an exercise tolerance test. Histological examination at 14 or 30 days after the transplantation was conducted by means of immunostaining and quantitative image analysis. An improvement in the functional status of the cardiovascular system was observed after both the autologous and the allogeneic transplantations. Location of the label-positive cells within the heart was restricted to the affected part of myocardium. The transplanted cells could give rise to fibroblasts or myofibroblasts but not to cardiac myocytes or blood vessel cells. Both types of transplantation positively influenced scarring processes, and no expansion of fibrosis to border myocardium was observed. Left ventricular wall thickening associated with reduced dilatation index was promoted by transplantation of the autologous cells. According to the results, multipotent stromal cell transplantation prevents adverse remodeling and stimulates left ventricular reverse remodeling.

## 1. Introduction

Since the first clinical trials of multipotent stromal cells (MSCs) transplantation in the nineties, more than 2,000 patients have been administered with allogeneic or autologous MSCs for the treatment of various conditions including cardiovascular diseases. Numerous preclinical studies and clinical trials have shown the potential and safety of MSC-based therapy; however, the therapeutic effects observed in clinical trials to date appear to be contradictory [1]. In the case of myocardium, this is due primarily to low rates of both survival and differentiation of MSCs to cardiac myocytes. However, these indicators were measured mainly for short periods after acute myocardial infarction, and only few studies are dealing with its long-term effects such as chronic heart failure and ischemic cardiomyopathy (e.g., [2]). In general, reported therapeutic impacts of MSC transplantation for the chronic consequences of myocardial infarction are subtle and understudied.

For that reason, many studies aimed to enhance the therapeutic effects of MSCs on cardiac conditions which are currently underway. Certain types of preconditioning or predifferentiation stimuli are shown to improve MSC survival, grafting, and ultimately the therapeutic effect of transplantation [3]. Another approach is to combine the MSC transplantation with some other mild supporting interventions, for example, targeted delivery of growth factors [4]. Despite the indisputable achievements in this field, a more refined concept of the basic mechanisms of MSC action would be helpful.

There are three functional hallmarks in myocardium repair potentially influenced by the MSCs: (1) strength and viability of cardiac myocytes (CMCs) regulated by prevention of cell death and/or promotion of cell replacement; (2) state of myocardial perfusion; (3) infarction scar genesis and remodeling. Enhanced perfusion of myocardium, especially of its hibernating parts, is necessary for effective tissue repair and improvement of heart function after ischemic damage, and stimulation of angiogenesis in the scar has a beneficial effect on myocardium regeneration, particularly on left ventricular reverse remodeling (LVRR). The dynamic scar rebuilding is believed to play a crucial role in LVRR [5].

Two main hypotheses for MSCs modes of action are discussed in the current literature [6–8]. The first hypothesis is dealing with replacement of dead CMCs and blood vessel cells with new counterparts derived from the graft by transdifferentiation (the “replacement mechanism”) [9, 10]. The second one involves various routes of regulation of local immune response and regeneration by paracrine factors produced by transplanted MSCs (the “paracrine mechanism”) [11, 12]. There is some evidence of MSCs transdifferentiation as confirmed by means of vital labeling and immunohistochemistry with differentiation-specific markers [13–15]. However, only few studies, for example [16], assess precise localization, morphology, and functional properties of labeled cells, follow transplantation. Furthermore, a possibility of cell fusion is sometimes difficult to exclude [17]. Another plausible mechanism of the enhancement, paracrine stimulation promoted by MSCs, is more substantiated [18, 19].

Effects of MSCs on myocardial repair have been assessed for animal models with a short time span (usually less than one week) between acute myocardial infarction (MI) and MSCs administration. Relatively few studies are published for MSCs transplanted after a longer delay, when the microenvironment of the infarction area essentially differs from that in the acute period (e.g., [20]). In this phase of recovery, the overall condition can be defined as a chronic heart failure due to ischemic cardiomyopathy. Here we report the analysis of delayed intracoronary infusion of MSCs for a rat model and describe LVRR caused by the autologous cell transplantation and probably supported by transdifferentiation of MSCs into fibroblasts and myofibroblasts.

## 2. Materials and Methods

### 2.1. Animals

Experimental work involving animals was carried out according to the rules of laboratory practice (National Guidelines number 267 by Ministry of Healthcare of the Russian Federation, June 1, 2003), and all efforts were made to minimize suffering. The study was approved by Ethical Review Board at the Institute for Human Morphology (Protocol number 4, March 12, 2010). In total, 85 Sprague-Dawley male rats weighing 180–200 g were used in the study. All animals underwent bone marrow aspiration from both tibias, and MI followed by 20-minute reperfusion was modeled. The animals were randomly allocated to experimental groups. At 30 days after the MI, intracoronary transventricular transplantation of MSCs was performed (5 × 10^6^ of autologous MSCs in 1 mL of saline for one experimental group, *n* = 29, and 5 × 10^6^ of allogeneic MSCs in 1 mL of saline for the other experimental group, *n* = 29). The control group animals (*n* = 27), also at 30 days after the MI, were infused with 1 mL of saline with no cells (sham transplantation). At posttransplantation (p/t) days 14 and 30 (i.e., resp., at 44 and 60 days after the MI), the animals underwent physiological examination and were consequently withdrawn from the experiment for morphometric and immunohistochemical examination of tissues.

### 2.2. MSCs Isolation and Culture

Bone marrow aspiration was performed immediately before MI modeling, that is, 30 days before transplantation. The procedure was performed under anesthesia with 120 : 10 mg/kg of ketamine : xylazine (Sigma-Aldrich Co. LLC, St. Louis, IL, USA), by puncture of spongy substance of both tibia bones through the knee joint cavity. Mononuclear cell fraction was isolated from bone marrow suspensions by the density gradient centrifugation [21]. The mononuclear cell suspensions were plated at density of 3000 cells/cm^2^ in culture dishes with DMEM/F12 1 : 1 medium supplemented with 10% fetal calf serum (FCS), 2 mM L-glutamine (PAA Laboratories GmbH, Pasching, Austria), and 0.5 mg/mL amikacin (Sintez JSC, Kurgan, Russia). The dishes were incubated at 37°C in humidified atmosphere with 5% CO_2_. The number of passages was limited to four to preserve the MSC phenotype and the multilineage differentiation capacity [22].

Expression of characteristic MSC surface markers was evaluated by flow cytometry. The cells of the 2–4 passage were detached from plastic with trypsin-EDTA solution, washed twice in PBS (pH = 7.4), resuspended in 0.1% FCS in PBS, incubated with phycoerythrin-conjugated antibodies to CD73, CD90, and CD105 (the MSC markers) and CD34, CD45, CD19, and CD11b (early and late hematopoietic stem cell markers, BD Biosciences, Franklin Lakes, NJ, USA) for 30 min, then washed, and analyzed using a flow cytometer (BD Biosciences, USA).

To confirm differentiation capacity of MSCs, cell cultures were plated on 60 mm Petri dishes and cultured until they reached confluence; by then the growth medium was replaced with differentiation medium from StemPro Adipogenesis Differentiation Kit, StemPro Osteogenesis Differentiation Kit, or StemPro Chondrogenesis Differentiation Kit (Life Technologies, Carlsbad, CA, USA). We demonstrated adipogenesis by staining with Sudan III, chondrogenesis by staining with 1% solution of alcian blue (pH = 2.5), and osteogenesis by staining with alizarin red S, pH = 4.1 (all manufactured by BioVitrum, Russia).

The MSCs of the third passage were labeled with PKH26 Red Fluorescent Cell Linker Kit (Sigma-Aldrich Co. LLC, USA) in accordance with the manufacturer's recommendations.

### 2.3. Modeling of MI with Postinfarction Cardiosclerosis

The thoracotomy was performed as described previously [23] under anesthesia with 120 mg/kg ketamine : 10 mg/kg xylazine (Sigma-Aldrich, USA) and mechanical ventilation with 7025 Rodent ventilator (Ugo Basile, Comerio, Italy) at 30% oxygen inspiratory concentration, 60–70 bpm respiratory rate, and 10 mL/kg tidal volume. The left descending coronary artery was ligated 3 mm below the left atrial appendage. The infarction was detected by visualization of a pale area with indistinct outline in the anterior and lateral myocardium of LV and confirmed by typical electrocardiogram changes in ST segment and QRS complex.

### 2.4. Intracoronary Transventricular Infusion of MSCs

In order to measure blood pressure and myocardial contractility indices and to infuse the MSCs, a catheter was implanted into the LV via the left and right carotid arteries 30 days after the MI. The procedure was performed under anesthesia with 120 : 10 mg/kg of ketamine : xylazine (Sigma-Aldrich, USA). The catheter was connected to electric pressure transducer DTX Plus TNF-R (Becton Dickinson, Franklin Lakes, NJ, USA). The MSCs were infused into the LV cavity against background of short aortic cross-clamping (1-2 sec) to ensure the transplant delivery into the coronary blood vessels rather than the systemic circulation. The aortic cross-clamp was performed through a small skin incision in the left lateral part of the chest over the sternum, with an L-shaped rod inserted into the incision over the aorta in the perpendicular plane. After the consequent rapid decline of the blood pressure, we infused the transplant (5 × 10^6^ of the MSCs in 1 mL of saline). In a similar way, the control group animals received 1 mL of saline with no cells (sham transplantation).

### 2.5. Exercise Tolerance Test

Swimming test was applied at two time points: before the transplantation and just before the animals were sacrificed. The rats were forced to swim and the duration of active swimming was registered.

Electrocardiograms were recorded at three consequent time points: during the MI modeling (to confirm infarction), immediately before the transplantation, during the transplantation to check for arrhythmia, and before the animals were withdrawn from the experiment. The recording was performed under anesthesia, along with the systemic arterial blood pressure and the LV pressure recording.

### 2.6. Morphometry

The animals were sacrificed in a CO_2_ chamber at p/t days 14 and 30 (i.e., at 44 and 60 days after the MI). The organs were partly fixed with formaldehyde and embedded in paraffin, partly cryofixed. Sections of the heart (5–7 *μ*m thick) were taken at 10 levels, from apex towards atriums, with 500 *μ*m spacing. Morphometric examination and cell migration analysis were performed with Axioplan 2 fluorescence microscope (Carl Zeiss, Oberkochen, Germany) at 400x magnification. The number of labeled cells was counted in 50 fields of view (50 × 37070 *μ*m^2^) for each organ per experimental group.

Morphometric examination of serial transverse sections of the heart, performed as described elsewhere [24], was based on the following measurements: scar tissue area; LV wall thickness in the scar zone; LV wall thickness in the border zone; interventricular septum wall thickness; LV cavity area. These values were used to calculate indices: scar size (%) = scar area/LV wall area∗100; LV dilatation index (%) = LV cavity area/total LV area∗100; hypertrophy index (%) = LV wall thickness in the border zone/interventricular septum wall thickness∗100; scar thickness (%) = LV wall thickness in the scar region/interventricular septum wall thickness∗100.

The measurements were made in 25x magnification panoramic images of serial cross-sections of the heart, spanning 10 levels from the apex to the atrium. Thus, 10 measurements of LV cavity area and related indices per animal were made. Taking into account that the orientation of the cut could deviate from the strict perpendicular, to define the dilatation index the LV cavity area was related to the total LV area (i.e., the cavity area + the LV wall area). The same morphometric scheme is used elsewhere [25, 26].

Mallory's trichrome stain (BioVitrum, Saint-Petersburg, Russia) was used to count blood vessels involved in actual blood supply. The assessment of angiogenesis by blood vessel number and volume density was restricted to the scar area. The measurements were performed for 100 randomly selected fields of view per group. To obtain the volume density, the total area of vascular lumens was divided by the total area of the image.

Picrosirius red stain (BioVitrum, Russia) was used to assess the scar maturity by polarized light microscopy. With the thick bundles consisting predominantly of type I collagen, visualized as red, and the thin bundles consisting predominantly of type III collagen, visualized as green, the ratio between corresponding areas was considered as an index of scar maturity [27]. The measurements were performed for 100 randomly selected fields of view per group.

The scar, the border zone, and the normal myocardium were distinguished by characteristic histological features. The border zone was defined conventionally as a transitional tissue formation between the scar and the normal myocardium, populated by live nucleated cardiomyocytes of normal shape and size, and filled with loose fibrous connective tissue of perimysium (i.e., the remote areas of intact cardiomyocytes with unaltered perimysium were taken for the norm). Such a distinction, despite being relative, is widely used in pathomorphology and the MRI diagnostics [28].

### 2.7. Immunohistochemistry

Both cryosections and paraffin sections were used in the study. Following hydration, the paraffin sections were treated consequently with citrate buffer (pH = 6.0 for 30 min at about 100°C), 3% hydrogen peroxide blocker, and 10% blocking solution (BioVitrum, Russia). Immunohistochemical assay was performed with antibodies to differentiation-specific and cell state-specific markers including connexin-43 (the marker of cardiac myocytes), CD34 (the marker of endothelial cells), *α*-SMA (*α*-smooth muscle actin, the marker of smooth muscle cells, and myofibroblasts), Fap*α* (the marker of reactive fibroblasts), CD68 (the marker of macrophages), and Ki67 (the marker of cell proliferation), all manufactured by Abcam, Cambridge, UK. Incubation with primary antibodies was carried out at 4°C overnight, in concentrations recommended by the manufacturer. The excess of primary antibodies was removed by three consequent washes with PBS; the sections were further incubated in 1 : 200 dilutions of FITC-conjugated secondary antibodies at room temperature for 1 hour. The sections were additionally stained with DAPI (Sigma Aldrich, USA) to label the nuclei. The number of positively stained cells was counted in 50 fields of view per experimental group.

### 2.8. Statistical Analysis

Statistical analysis was performed with SigmaPlot 11.0 software. Mean and standard deviations, confidence intervals, median values, and percentiles were calculated for all variables within groups. The data are presented in histograms as mean ± standard error of the mean (SEM) unless otherwise specified. The absolute values were compared using Student's *t*-test for normally distributed data. In cases of a distribution different from normal, the Mann-Whitney test was applied. The percent values were compared using Kruskal-Wallis rank single-factor dispersion analysis. The differences with *P* < 0.05 were considered significant.

## 3. Results

### 3.1. Characterization of MSCs

**Figure 1:**
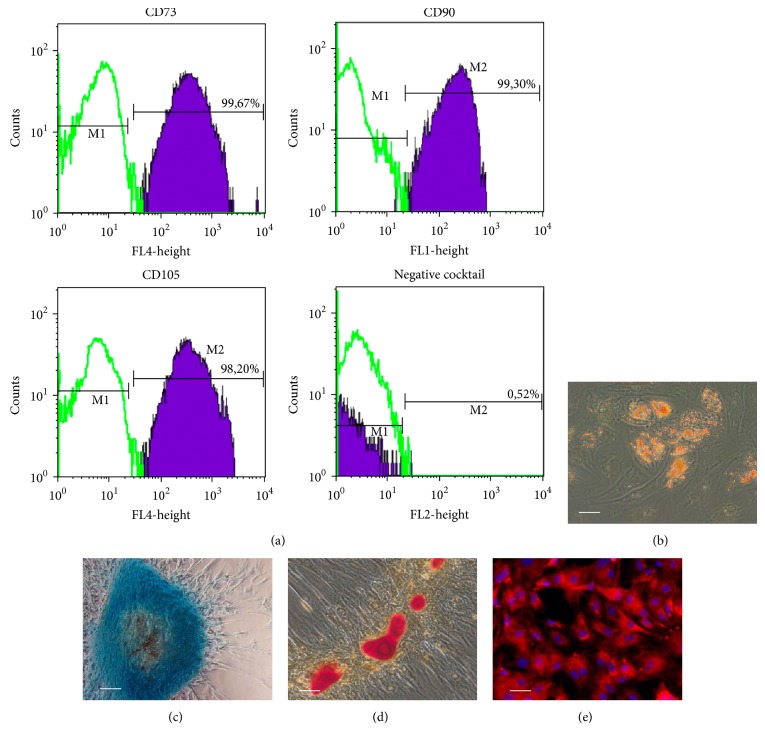
Flow cytometry and induced differentiation analyses of MSCs. (a) The cultures were positive for CD73, CD90, and CD105 but negative for CD34, CD45, CD19, and CD11b. (b) Appearance of intracellular lipid-rich vacuoles during MSCs adipogenic differentiation was confirmed by Sudan III staining at day 14. (c) Differentiation of MSCs to chondrogenic lineage was visualized by staining for mucopolysaccharides with alcian blue at day 28. (d) Differentiation of MSCs to osteogenic lineage was visualized by staining for calcifications with alizarin red at day 21. (e) Microscopic evidence for effective labeling of MSCs with PKH26 (the nuclei are additionally stained with DAPI). Scale bars, 50 *μ*m.

Cell cultures derived from rat bone marrow stroma were composed of adhesive cells capable of growth on untreated plastic. The cells were positive for CD73, CD90, and CD105 but negative for CD11b, CD19, CD34, and CD45 (Figure 1(a)). The cultures entered adipogenic, chondrogenic, or osteogenic differentiation under appropriate stimuli. The lipid droplets accumulated in the cytoplasm starting from days 5–7 of adipogenic induction (Figure 1(b)); the mucopolysaccharides or calcium deposition started after 3 weeks of incubation in the chondrogenic or osteogenic differentiation media (Figures 1(c) and 1(d), resp.). Thus, the cells showed full consistency with the minimal criteria for MSCs [29].

After labeling with PKH26, the red fluorescence of membranes with the peak emission at 567 nm was observed in all cells (Figure 1(e)). The substance did not interfere with the proliferation capacity; the fluorescent particles were distributed between the daughter cells in the course of cell division and no excretion of the label into environment was detected.

### 3.2. Heart Function Recovery Related to the MSCs Transplantation

The average mortality constituted 17.8, 18.2, and 18.6% for, respectively, the sham (saline), the autologous MSCs, and the allogeneic MSCs transplantation; the between-group differences in the mortality were insignificant (*P* > 0.05 by *z*-test). The protocol of forced swimming test was modified to reflect the weakened function of the cardiovascular system; it was a variation of exhaustive swim test protocol in which the cessation of the active movement, not the drowning, was taken for an endpoint. The swim duration after the sham infusion did not differ from the values for 14 or 30 days after the MI; on the contrary, significant increases were observed after both the autologous and the allogeneic MSC transplantations (Table 1).

For all groups, we found increases in the arterial blood pressure, the maximum LV pressure, and the contractility index, +*dp*/*dt*. According to [30], these changes may indirectly indicate the recovery of the heart inotropic function. For the control group, increased values were recorded at p/t day 30, while the experimental groups showed some quicker increases. In the allogeneic MSCs transplantation group, the LV contractility index was increased for both p/t day 14 and p/t day 30 time points. The autologous MSCs transplantation resulted in an increased LV maximum pressure at p/t day 14. However, in the autologous MSCs transplantation group the LV contractility index remained unchanged at p/t day 14 (*P* > 0.05), and it was significantly lower than that in the control group at p/t day 30.

### 3.3. Homing and Differentiation of Transplanted MSCs

**Figure 2:**
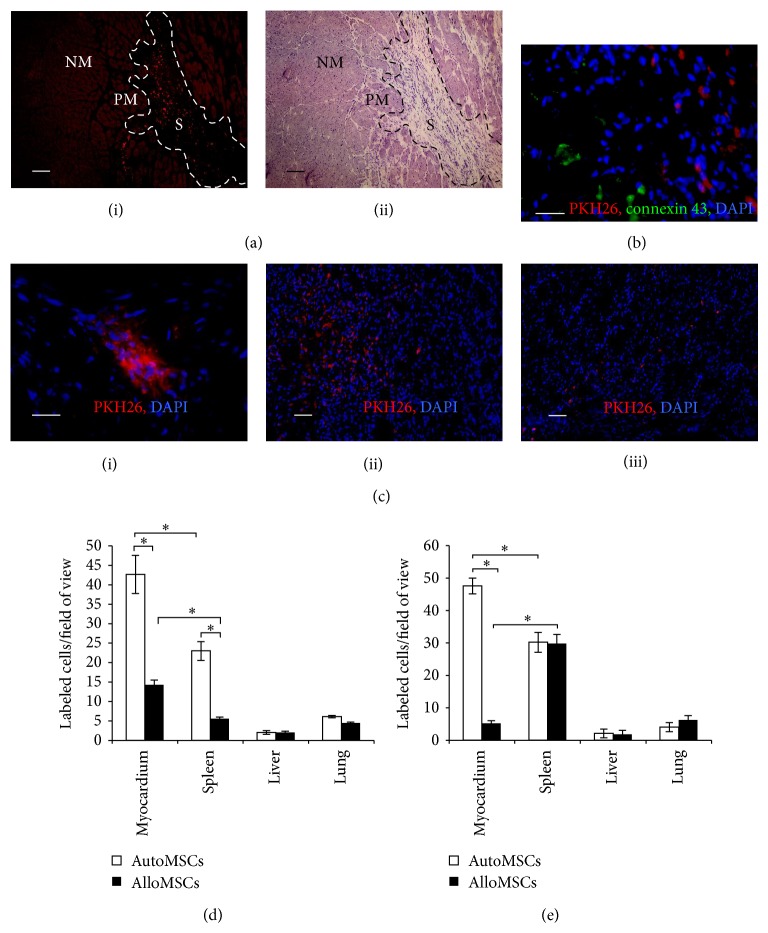
Homing of MSCs following intracoronary infusion. Fluorescence and light microscopy of heart cryosections following the autologous transplantation. (a) The red fluorescent cells are observed only in the affected myocardium (the areas are designated S for scar, NM for normal myocardium, and PM for perifocal myocardium (border zone)). (b) The labeled cells are not found in the border myocardium in the vicinity of cardiac myocytes (connexin-43-positive, green). (c) Typical images subject to quantitative analysis: (i) 10 min after infusion the labeled autologous MSCs are attached to the inner surface of vascular wall; (ii) at p/t day 14 (ii) the autologous MSCs and (iii) the allogeneic MSCs are found in the scar only. The diagrams show numbers of the label-presenting cells in heart, spleen, and liver counted in 37070 *μ*m^2^ field of view (d) at p/t day 14 and (e) at p/t day 30. The data are presented as mean ± SEM with asterisks indicating significant differences. Scale bars: (a), 100 *μ*m; (b) and (c)i, 25 *μ*m; (c)ii and (c)iii, 50 *μ*m.

**Figure 3:**
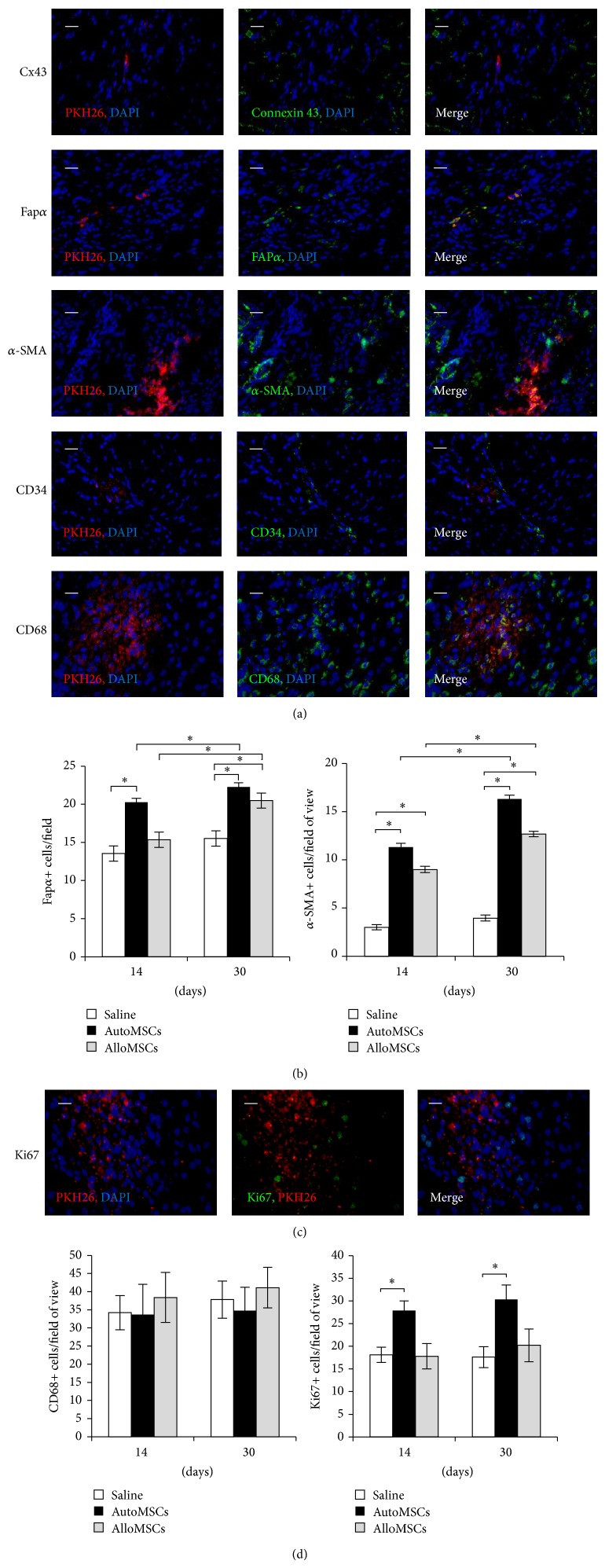
Differentiation, viability, and elimination of the transplanted MSCs. (a) Fluorescence microscopy of transverse cryosections of the heart at p/t day 14. None of the transplanted MSCs differentiated into cardiac myocytes, endothelial cells, or smooth muscle cells of blood vessels. Some label-presenting cells outside the blood vessels were positively stained with antibodies specific to *α*-SMA or Fap*α*. (b) Cell counts of reactive fibroblasts and myofibroblasts in the scar. (c) Fluorescence images of transverse cryosections of the heart at p/t day 14. Some of the label-presenting cells are positively stained with antibodies to Ki67 or CD68. (d) Counts of Ki67+ and CD68+ cells in the scar. The data are presented as mean ± SEM with asterisks indicating significant differences. Scale bars, 25 *μ*m.

The MSCs infused into the LV cavity simultaneously with the aortic cross-clamp successfully reached the right and left coronary arteries. In three animals, that died 5 to 10 minutes after transplantation, the labeled cells were found uniformly distributed in the coronary vessels of all cardiac tissues adhering to the blood vessel walls (Figure 2(c)i). At p/t days 14 and 30, the presence of the labeled cells was restricted to the infarction area (Figures 2(a) and 2(b)). Moreover, the labeled cells were found mostly in the postinfarction scar, not in the border zone (Figures 2(a), 2(b), 2(c)ii, and 2(c)iii). The labeled cells displayed a fibroblast-like phenotype, contained undamaged nuclei, and were located among the collagen fibers. Positive staining of some of the labeled cells with Ki67-specific antibodies confirmed the cell viability and proliferation activity (Figure 3(c)). Totally, the numbers of Ki67-positive cells after the autologous transplantation were higher than those in the other groups (Figure 3(d)).

Colonization of the scar with the transplanted material was significantly more intense for the autologous MSCs than the allogeneic MSCs, as estimated by cell counts (42.7 ± 4.9 and 14.2 ± 1.2 labeled cells per field of view, resp.). In the autologous transplantation group, the labeled cell counts in the scar between p/t days 14 and 30 remained constant (42.7 ± 4.9 and 47.6 ± 2.4, *P* > 0.05), while in splenic tissue they rose to 30.2 ± 3.1. In the allogeneic transplantation group, the labeled cell counts in the scar between the time points decreased from 14.2 ± 1.2 to 5.2 ± 0.9 (*P* < 0.05), and for splenic tissue the counts rose from 5.6 ± 0.5 to 29.8 ± 2.8 (*P* < 0.05).

The diagrams in Figures 2(d) and 2(e) show that the most rapid colonization of the scar with the transplanted cells was observed for the autologous MSCs. Some single red fluorescent cells were observed in lungs and livers of the autologous transplantation group animals at both p/t days 14 and 30. Much fewer label-presenting cells were found in tissues of the allogeneic transplantation group animals. This may indicate higher rates of elimination of the allogeneic cells by host immune system. Staining with CD68-specific antibodies confirmed partial elimination of both allogeneic and autologous MSCs by host macrophages (Figure 3(c)). At all thus, histological examination revealed no signs of transplant rejection, and estimated levels of macrophage infiltration were the same for all groups (Figure 3(d)).

### 3.4. Differentiation of MSCs following Transplantation

The presence of the labeled cells inside the heart tissues was restricted to the scar regardless of the transplant origin (autologous or allogeneic MSCs, Figure 2). As revealed by fluorescence microscopy, few cells inside the scar were label-positive ones, and these were morphologically indistinguishable from surrounding cells. No CMCs with the label in their membrane or cytoplasm were found in border zone and unaffected parts of myocardium. In the border zone, labeled cells were occasionally found in connective tissue next to CMCs. Reciprocally, none of the label-presenting cells was stained with connexin-43-specific antibodies (Figure 3(a)).

In both experimental groups, the labeled cells were absent from tunica intima and tunica media of blood vessels of the scar (Figure 3(a)). Only the adventitia contained the labeled cells, but in this case it was difficult to distinguish the adventitia cells from the scar fibroblasts. None of the label-presenting cells was stained with CD34-specific antibodies. This suggests that the transplanted cells are not directly involved in the formation of new blood vessels in the scar and do not differentiate into endothelial cells. No label-presenting cells were integrated into vessel walls in the unaffected myocardium as well.

The cryosections with fluorescent labeled cells were stained with an antibody to alpha smooth muscle actin (*α*-SMA), the marker of smooth muscle cells of the vascular tunica media (Figure 3(a)). No label-presenting cells were found among that population. We observed, however, that the antibody reacted not only with the vascular smooth muscle cells but also with the fibroblast-like cells of the scar. These cells were identified as myofibroblasts, a special type of fibroblasts that are able both to contract and to produce the extracellular matrix [31, 32]. Superimposition of the fluorescence images revealed a subpopulation of label-positive myofibroblasts (Figure 3(a)).

Taking into consideration the localization and morphological features of this subpopulation, we hypothesized that the MSCs differentiated into the scar myofibroblasts. A further immunohistological assay was performed with the antibody specific to the molecular marker of reactive fibroblasts, Fap*α* [33]. By definition, fibroblasts of this type emerge in wound healing, granulation tissue formation, and certain sarcomas; they actively produce Fap*α* homodimers [34]. Large numbers of the Fap*α*-positive cells were observed in the scars of both experimental groups and control group (Figures 3(a) and 3(b)). Apparently, these cells were active fibroblasts secreting collagen and actively participating in the scar formation. Moreover, many of the label-presenting cells were also Fap*α*-positive ones. (This may reflect differentiation of the transplanted cells into reactive fibroblasts.) The double-positive fractions of the label-presenting cells were similar for the autologous and the allogeneic transplantation groups (28.7 ± 6.4% and 26.3 ± 7.8, *P* > 0.05).

### 3.5. The Postinfarction Scar Morphology and Cellular Composition

**Figure 4:**
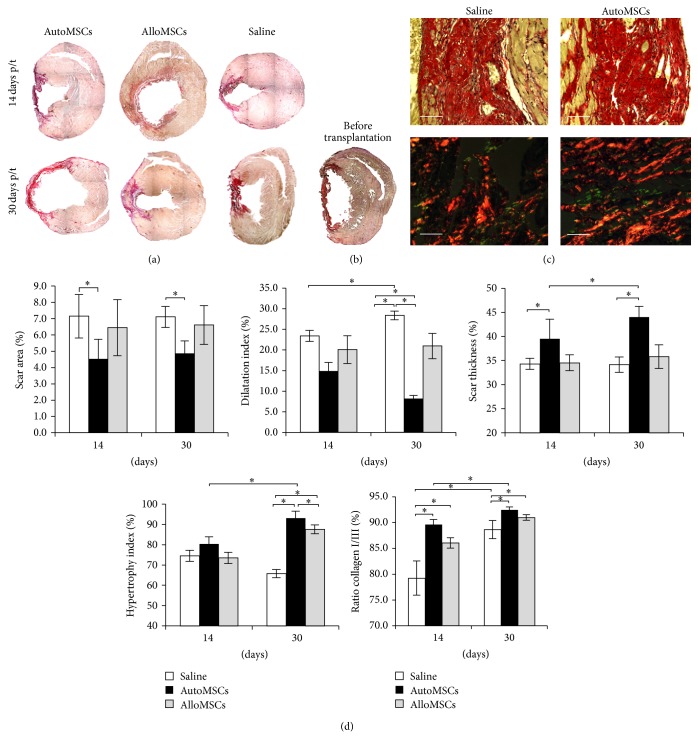
The scar morphology and LV remodeling features related to the transplantation. (a) Transverse sections of the heart stained with picrosirius red. Large foci of postinfarction cardiosclerosis were found in all animals including the controls at p/t day 14 (i.e., at 44 days after the MI); panoramic shots were made at 25x magnification. (b) Transverse section of the heart before transplantation, 30 days after MI. (c) Fine structure of the scar, stained with picrosirius red, observed in normal and polarized light. The scar of the autologous transplantation specimen appears more organized and compact. (d) The diagrams show dynamics of the indices relevant to LVRR. The data are presented as mean ± SEM with asterisks indicating significant differences. Scale bars, 50 *μ*m.

At p/t days 14 and 30 (i.e., resp., 44 and 60 days after MI), the large areas of postinfarction cardiosclerosis in the anterior and lateral walls of LV were observed for all groups (Figure 4(a)). The scar consisted mainly of thick collagen fiber bundles with rare spindle-shaped cells in between. Boundaries between the cardiac fibrosis and the border zone were vague, with collagen fiber bundles intertwining with muscle fibers at the transition. Several animals manifested the chronic aneurysm of the LV, and we observed a marked myocardial hypertrophy along the boundaries of the aneurysms or the scars. Conversely, thinning of the LV wall accompanied by dilatation of LV cavity was observed in several animals of the control group. The CMCs in the border zone were often binucleated and hypertrophic. The muscle fibers were surrounded by widened perimysium with numerous blood vessels of different diameter (Figure 4(a)).

In a later period after the MI, we found cells abundantly pervading the scar in the form of diffuse agglomeration of mononuclears. High cell densities especially, in combination with the more compact extracellular matrix consisting of longitudinal, regularly oriented thick collagen fiber bundles, were observed for the autologous MSCs transplantation (data not shown). In the control group, the structure of scar tissue was the loosest (Figure 4(d)).

The counts of reactive fibroblasts (i.e., Fap*α*-positive cells) were maximal for the autologous MSCs at p/t days 14 and 30 (Figure 3(b)). Allogeneic MSCs transplantation resulted in an increased amount of reactive fibroblasts at p/t day 30 only, and no changes in the reactive fibroblast counts were found for the control group (Figure 3(d)). The counted numbers of myofibroblasts (i.e., *α*-SMA-positive cells) were the highest for both autologous and allogeneic MSCs transplantation at both 14 and 30 day time points, while the counts of myofibroblasts for the control group remained constant (Figure 3(b)).

Scar formation generally involves an increase in amount of collagen fibers and a positive shift in collagen type I to collagen type III ratio [35–39]; the latter can be used as a scar tissue maturity indicator [27]. The highest values, similar for all three groups, were recorded for p/t day 14 (Figure 4(d)). The values for p/t day 30 were similar too, especially between the two experimental groups. Perhaps, the scar maturation was completed before p/t day 14 (i.e., before postinfarction day 44).

### 3.6. LV Remodeling

At 30 days after the MI (before the transplantation), all of the animals manifested postinfarction cardiosclerosis, with the scar size, the LV dilatation index, and the scar thickness constituting, respectively, 6.6 ± 0.8%, 21.3 ± 3.1%, and 27.4 ± 5.6%. Encouraged by the indications of the homing and differentiation of the transplanted cells into functionally active cells within the scar, we assumed that the transplantation can prevent adverse remodeling and even stimulate reverse remodeling. At p/t day 14 (i.e., 44 days after the MI), the scar dimensions were similar in all groups (Figure 4(d)). At p/t day 30, the scar dimensions in the autologous transplantation group were significantly smaller than those in the control group.

Dilatation index is an integrated parameter reflecting adverse LV remodeling, as it depends on both the size and thickness of the scar and the degree of the border myocardium hypertrophy [40]. Progressive adverse remodeling in the controls was revealed by a higher dilatation index compared to the experimental groups at p/t day 14 and aggravation of this trend at p/t day 30, as shown in Figure 4(d). Accordingly, the autologous MSCs transplantation resulted in reduced LV dilatation at p/t day 14 as compared to the controls. A further improvement in this group was detected at p/t day 30; meanwhile, the allogeneic transplantation had much more limited influence on the LV dilatation (Figure 4(d)). The affected ventricular wall thickness was increased at p/t day 30 in the autologous transplantation group, but we did not observe that in the control and the allogeneic transplantation groups (Figure 4(d)). Thus, the LVRR was observed only for the autologous transplantation. In both experimental groups, the transplantation resulted in apparent hypertrophy of the border myocardium beginning between p/t days 14 and 30 (Figure 4(d)).

### 3.7. Angiogenesis Induction by the Transplant

**Figure 5:**
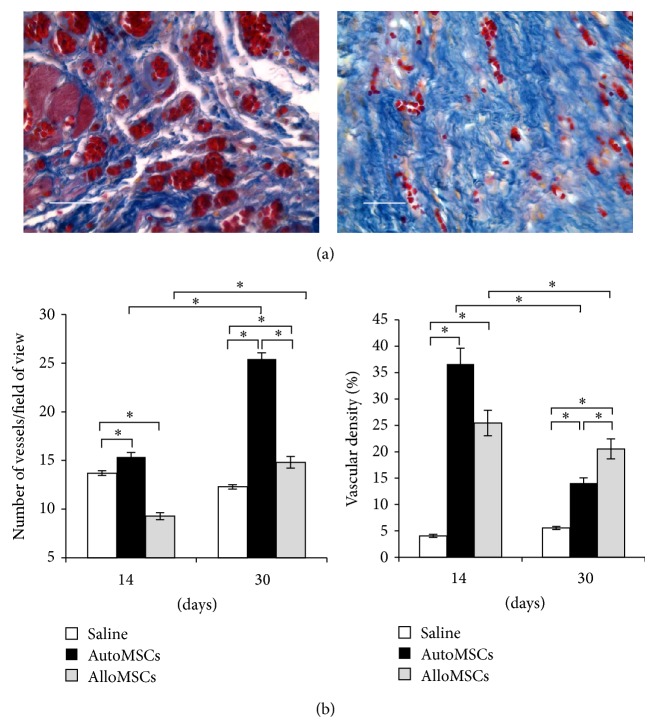
Enhanced angiogenesis associated with the transplantation. (a) Transverse sections of the heart at p/t day 14 stained with Mallory's corresponding to the autologous MSCs transplantation (left panel) and the control (right panel). (b) The diagrams show dynamics of relevant variables. The data are presented as mean ± SEM with asterisks indicating significant differences. Scale bars, 50 *μ*m.

None of the data indicated differentiation of transplanted cells into endothelium or smooth muscle cells (Figure 3(a)); nevertheless, the autologous transplantation resulted in a larger number of blood vessels at p/t day14 as compared with other groups (Figure 5(a)). At p/t day 30, the number of blood vessels for both experimental groups exceeded that for the controls (Figure 5(b)). The volume density evaluation is sometimes considered more appropriate than the blood vessel counts given that an enlargement of vessels is often coupled with a decrease in their number [41]. At p/t day 14, the volume density of blood vessels in the postinfarction scar was higher for both experimental groups than for the controls (Figure 5(b)).

## 4. Discussion

In this study, we infused the cells into coronary circulation at 30 days after the acute myocardial infarction, that is, when the scar formation has been completed, and on the background of the adverse remodeling of the left ventricle. We expected that the MSCs would settle in the border myocardium (border zone) and differentiate into cardiomyocytes or blood vessel cells, but these expectations proved wrong (which is consistent with the results for similar models described elsewhere [42, 43]). Instead of being assimilated by regenerating heart muscle, the transplanted cells settled in the scar and participated in its remodeling. This effect may be explained by chemoattraction exerted towards MSCs by the fibrotic tissue on later stages of the scar formation. Differentiation of the MSCs to fibroblasts and myofibroblasts inside the scar is obviously promoted by the scar microenvironment.

We modeled postinfarction cardiosclerosis to study the influence of the MSCs transplantation on the remote terms after acute myocardial infarction. Essentially, we modeled not the regeneration of myocardium following the acute myocardial infarction but the chronic heart failure due to ischemic cardiomyopathy. It is primarily associated not with the inflammation or heart muscle reparation indices but with the adverse remodeling of left ventricle which is manifested by morphological changes in the scar and the border myocardium. Postponement of the transplantation to 30 days after the acute myocardial infarction is a known approach to model MSCs action in chronic heart failure due to ischemic cardiomyopathy [42, 44].

The proposed method of transventricular intracoronary cell transplantation requires no radiological monitoring of the catheter localization and provides efficient cell delivery to the heart of small laboratory animal. The intracoronary delivery is admittedly optimal as it has the same efficiency as intramyocardial delivery but is much less invasive [45]. Consequent localization of the transplanted cells indicates their homing to the affected zone.

The transplanted cells retained their viability throughout the observation period, as indicated by the active homing, normal cellular morphology, and observable mitotic activity confirmed by expression of Ki67 by labeled cells. Some part of the MSCs entered systemic circulation and consequently settled in the hematopoietic tissues, for example, red pulp of the spleen. According to the results, in the autologous transplantation group the labeled cell numbers in the scar between p/t days 14 and 30 remained constant, while in the allogeneic transplantation group they decreased, and the counts for splenic tissue rose significantly in both groups. For the allogeneic MSCs, the decrease in myocardium and the increase in spleen at 30 days p/t can be explained by cell death and consequent elimination by macrophages, probably migrating to the spleen, and other blood-forming organs. Label-positive macrophages (defined as CD68+ cells) were observed inside the scar equally in all groups. It is plausible that we observed accumulation of macrophages, loaded with the label as a consequence of phagocytosis of the whole MSCs or their fragments, in the spleen. Independently of their origin (autologous or allogeneic), the cells were partly eliminated by host immune system; however, histological examination revealed no signs of transplant rejection, and the extent of macrophage infiltration was the same for all groups including the controls.

One of the most important questions in studying the role of MSCs in myocardium regeneration is whether these cells are able to differentiate into specialized cells of the heart. The capability of MSCs to differentiate into CMCs, endothelial cells, or smooth muscle cells has been demonstrated* in vitro* [46–48]. However, there is no evidence of such differentiation* in vivo*, and its possibility is still under discussion [49]. Studies demonstrating transdifferentiation of MSCs into specialized cells of the heart are based on using differentiation-specific markers [50, 51]; obviously, supporting criteria of MSCs differentiation into specialized cell types should also include reliable indicators of cellular localization and morphology. In these terms, the current study provides no support to differentiation of the MSCs into CMCs or blood vessel cells* in vivo*; the absence of red fluorescence in CMCs also excludes any fusion between them.

By design of the study, the MSCs were transplanted at 30 days after the MI, that is, at the peak of myocardial scarring. By this time, the inflammation ceases and the majority of cells in the affected zone of myocardium are constituted by fibroblasts and myofibroblasts, the key cell types in the formation of the granulation tissue and subsequently of the scar [35, 37]. At earlier stages, myofibroblasts actively synthesize the extracellular matrix and contract the scar preventing the expansion of the affected zone; at later stages, these cells provide thickening and strengthening of the infarcted ventricle wall. Presumably, stimulated by the scar microenvironment, the transplanted MSCs differentiated into fibroblasts and myofibroblasts, as some of them were positively stained with antibodies to Fap*α* and *α*-SMA.

When myofibroblasts migrate to the border and intact myocardium, they stimulate the expansion of fibrosis [31]. Paradoxically, the same cells can both prevent and promote adverse LV remodeling. The outcome depends on the application point of transplanted cell activity; the MSCs prevent adverse remodeling in the scar, while in the intact myocardium they stimulate it. The adverse remodeling is caused by expansion of the scar tissue. It is mediated by an invasion of the border and intact zones of myocardium by myofibroblasts. These relocated myofibroblasts proliferate and produce the extracellular matrix [32, 35]. With the MSCs transplanted at the correct stage of the recovery, the consequent increase in the number of reactive fibroblasts and myofibroblasts in the scar results in the scar thickening and strengthening without expansion of the fibrosis to the border myocardium. In this study, particularly favorable trends in the LV remodeling were shown for the autologous transplantation. These included the increased thickness of the infarcted LV wall accompanied by the enhanced scar maturity as judged by the increased thickness and regular orientation of the collagen fibers.

Another variable used to describe adverse remodeling is the LV dilatation index. It is closely associated with the LV wall thickness and tension. According to the law of Laplace, the LV wall tension is directly proportional to the inner pressure and radius of LV cavity and is inversely proportional to the LV wall thickness [52]. The LV wall thickening results from both hypertrophy and scar thickening; leading to a decrease in LV tension with a consequent decrease in LV dilatation, and it ultimately stimulates the LVRR.

The postinfarction scar remodeling is believed to be regulated via dynamic changes in the extracellular matrix, basically composed of collagen fibers. Controlled balance between the synthesis and degradation of collagen is supposed to promote the formation of a dense scar and at the same time to restrain the expansion of fibrosis to unaffected parts of myocardium [36]. In these terms, scarring may be defined as an adaptive response that provides structural and functional compensation to limited regenerative potential of myocardium itself. Enhancement of this response with MSCs may specify an independent concept for antiremodeling therapy.

The postinfarction scar is a dynamic formation, and a well-developed blood supply is necessary for its remodeling and adaptation to the new conditions of circulation and heart functioning [53, 54]. Transplantation of the MSCs, especially autologous, resulted in stimulation of angiogenesis that could contribute to the scar thickening and the hypertrophy of border myocardium. Although the transplanted cells did not appear to differentiate into endothelial cells or smooth muscle cells, the number of blood vessels and their volume density in the scar increased after the MSCs transplantation.

For the quantitative assessment of the angiogenesis, it is necessary to evaluate both of the indices, because an increase in the size of the vessels may be linked to a decrease in their counts in a field of view. An increase in total blood vessel area without any increase in their counts, which is typical for the early stages of angiogenesis, means an increase in volume density of newly formed blood vessels indicating higher perfusion rates of the area. The volume density of blood vessels can be inversely related to their number. In the granulation tissue angiogenesis, rapidly forming thin-walled capillaries and venules with an expanded lumen contribute in the area more than the arterioles that appear later [55].

The volume densities of blood vessels in the scar at p/t day 30 for the experimental groups were higher than those for the control group, but, compared with day 14, they were considerably (and significantly) decreased. This may indicate maturation of the primary blood vessels (i.e., capillaries and venules) accompanied by formation of the arterial vessels on later stages. Finally, the counts of arteries and arterioles in the maturing scar increase, whereas their total area is known to be considerably less than that of veins and venules.

The stimulation of angiogenesis and the hypertrophy of border myocardium after both the autologous and the allogeneic MSCs transplantations probably resulted from paracrine induction of reparative processes by the transplant. Stem or stem-like cells from various sources produce cytokines and growth factors that regulate regeneration. Various paracrine factors stimulate angiogenesis (VEGF, Ang I, FGF, PDGF, etc.), inhibit apoptosis (HGF, IGF-I), stimulate bone marrow-derived cell homing (SDF-1) and immobilization (GM-CSF), and regulate scar remodeling (MMP-3, MMP-6); paracrine factors can also induce border myocardium hypertrophy and exert an anti-inflammatory and immunomodulatory action [18]. Induction and modulation of reparative processes by paracrine factors, expressed by the transplanted cells, is considered very important [19, 56]. This component was intentionally left out of the study not to overload the work methodically; despite that, its importance was implied.

High levels of vascular endothelial growth factor (VEGF) expression are characteristic for MSCs both* in vitro* and* in vivo* [57]. Delivery of VEGF to myocardial infarction tissue (by immunoliposomes targeting overexpressed P-selectin) provided an exceptional support for MSCs (injected consequently into myocardium). The combination treatment increased blood vessel density and decreased collagen content in post-MI tissue. Besides, it significantly increased the engraftment of MSCs, with the engrafted cells contributing to the restoration of blood vessels [4]. Like many other proangiogenic factors, VEGF can be potently induced by hypoxia via HIF-1*α* pathway, and this may serve as a way to fine-tune its expression [58]. In particular, a specific VEGF-dependent enhancement of regeneration by hypoxia-preconditioned MSCs was shown for the rat massive hepatectomy model [59].

Due to its limited perfusion, the postinfarction scar represents a relatively hypoxic microenvironment (compared with the intact myocardium). This may attract the MSCs and promote their participation in the angiogenesis, plausibly via HIF-1*α*-mediated VEGF expression. A further, prolonged stay in the hypoxic microenvironment of the scar can shift the balance promoting differentiation of MSCs to myofibroblasts. Such a scheme works in chronic kidney disease, where a transition to fibrosis is shown to be triggered by hypoxia and mediated by conversion of pericytes into myofibroblasts [60]. Immediately after transplantation, MSCs are shown to continue producing VEGF along with some other proangiogenic factors [61, 62].

Stromal cell-derived factor 1 (SDF-1) signaling may give a major contribution not only to initial recovery from the damage, but also to extended processes of the scar remodeling. In myocardium, the intrinsic SDF-1 expression is upregulated immediately after myocardial infarction and downregulated within 7 days [63]. Reciprocally, MSCs express SDF-1-specific receptor CXCR4. Moreover, they express the IL8-specific receptors CXCR1 and CXCR2, the MIP-1a-specific receptor CCR1, and some other chemokine-specific receptors that may participate in the active homing of the cells to the damaged area [64]. It is demonstrated that the local trophic effects of MSCs on the myocardium require CXCR4 expression by cardiac myocytes and their progenitor cells and that these effects are exerted by MSCs, at least partly, through SDF-1 secretion. [65]. Also, for the burn wound healing model, overexpression of SDF-1/CXCR4 is shown to upregulate the mobility of mononuclear peripheral blood CD14+ cells, promoting their migration from bloodstream to wound sites [66]. The same interaction of SDF-1/CXCR4 may contribute to the trafficking of transplanted cells in an ischemic brain functional recovery model with postponed time of cell transplantation [67]. Perhaps some related circuit is also valid in our model, but this question is beyond the scope of this study.

## 5. Conclusions

Intracoronary infusion of the MSCs against the background of postinfarction cardiosclerosis at 30 days after MI leads to concentration of transplanted cells in the affected area of myocardium. Subsequent differentiation of transplanted cells into fibroblasts and myofibroblasts is highly probable. The infusion of autologous MSCs leads to strengthening of the scar without its expansion into the border myocardium, ultimately resulting in the LVRR and consequent improvement of the heart function. The transplantation of allogeneic MSCs is less efficient although it also accelerates the scar maturation, stimulates the angiogenesis within the scar, and induces the border myocardium hypertrophy. However, the infusion of allogeneic MSCs does not result in the LVRR.

## Figures and Tables

**Table 1:** The dynamics of cardiac function variables before and after the transplantation, mean ± SEM.

	Before infusion			14 days			30 days		
	Saline	AutoMSCs	AlloMSCs	Saline	AutoMSCs	AlloMSCs	Saline	AutoMSCs	AlloMSCs
Tolerance, min	20 ± 2	17 ± 1	17 ± 1	15 ± 3^*^	25 ± 2^∗#^	23 ± 2^∗#^	16 ± 3	30 ± 3^∗#&^	27 ± 1^∗#&^
HR, hb/min	199 ± 17	214 ± 10	212 ± 17	231 ± 8	224 ± 20	233 ± 14	216 ± 11	237 ± 10	198 ± 22
Average BP, mm Hg	77 ± 4	88 ± 6	82 ± 4	79 ± 2	83 ± 3	78 ± 2	95 ± 4^∗&^	84 ± 2	76 ± 2
LV BP max., mm Hg	84 ± 5	82 ± 7	79 ± 6^#^	110 ± 3	108 ± 5	102 ± 1^*^	116 ± 3^∗&^	108 ± 3^*^	97 ± 4^#^
+*dp*/*dt*	4128 ± 798	3725 ± 211	3913 ± 420	7112 ± 624	7523 ± 238^*^	5824 ± 72	7781 ± 364	6829 ± 348^*^	5534 ± 397^#^
−*dp*/*dt*	2396 ± 438	2220 ± 215	2330 ± 261	4598 ± 290^*^	4025 ± 567^*^	3816 ± 72^*^	4748 ± 353^*^	4638 ± 220^*^	3653 ± 279^*^

^*^ *P* < 0.05 as compared to initial values.

^#^ *P* < 0.05 as compared to the controls.

^&^ *P* < 0.05 as compared to p/t day 14.
